# 4090 Dysregulation of Skeletal Muscle Mitochondrial Function following Critical Illness: a Translational Approach

**DOI:** 10.1017/cts.2020.296

**Published:** 2020-07-29

**Authors:** Luther Gill, Liz Simon, Patricia Molina

**Affiliations:** 1 LSUHSC School of Medicine- LA CaTS; 2 LSU Health Science Center

## Abstract

OBJECTIVES/GOALS: The objective of the study was to determine whether CLP altered genes associated with mitochondrial function in the diaphragm. METHODS/STUDY POPULATION: A rodent cecal-ligation and puncture (CLP) model used to mimic sepsis-induced critical illness. The CLP model involved ligation of 50% of the cecum below the ileocecal valve in adult C57BL6 mice, followed by needle puncture of the cecum resulting in mid-grade sepsis. Mice survived for 48 hours or more, following injury. Diaphragm and limb muscles were harvested 24 hours following CLP (N = 6) and following a sham CLP procedure (N = 6). RESULTS/ANTICIPATED RESULTS: Gene expression of mitochondrial related genes (mef2c, myh1, pgc1-α), were significantly decreased in the diaphragm of CLP injured animals when compared to controls. In addition, ubiquitin ligases, genes associated with skeletal muscle atrophy murf1 and atrogin were increased in the diaphragm 24 hours after injury (p< 0.01). DISCUSSION/SIGNIFICANCE OF IMPACT: Our results indicate that sepsis-induced critical illness significantly impacts the expression of genes implicated in mitochondrial homeostasis and atrophy. Ongoing studies will identify whether CLP injury decreases skeletal muscle mitochondrial function.

